# Uncovering EEG Correlates of Covert Attention in Soccer Goalkeepers: Towards Innovative Sport Training Procedures

**DOI:** 10.1038/s41598-020-58533-2

**Published:** 2020-02-03

**Authors:** Camille Jeunet, Luca Tonin, Louis Albert, Ricardo Chavarriaga, Benoît Bideau, Ferran Argelaguet, José del R. Millán, Anatole Lécuyer, Richard Kulpa

**Affiliations:** 1 CLLE Lab, CNRS, University Toulouse Jean Jaurès, Toulouse, 31000 France; 2 Inria, University Rennes, IRISA, CNRS, Rennes, 35000 France; 3 Ecole Polytechnique Fédérale de Lausanne (EPFL), Geneva, 1202 Switzerland; 4 Intelligent Autonomous Systems Lab, Department of Information Engineering Universitá degli Studi di Padova, Padova, 35131 Italy; 5 Campus Biotech, Geneva, 1202 Switzerland; 6 ZHAW Datalab, Zürich University of Applied Sciences, Winterthur, 8400 Switzerland; 7 University Rennes, Inria, M2S - EA 7470, Rennes, 35000 France; 8 Dept. of Electrical and Computer Engineering, The University of Texas at Austin, Austin, 78712 USA; 9 Dept. of Neurology, The University of Texas at Austin, Austin, 78712 USA

**Keywords:** Attention, Brain-machine interface, Human behaviour

## Abstract

Advances in sports sciences and neurosciences offer new opportunities to design efficient and motivating sport training tools. For instance, using NeuroFeedback (NF), athletes can learn to self-regulate specific brain rhythms and consequently improve their performances. Here, we focused on soccer goalkeepers’ Covert Visual Spatial Attention (CVSA) abilities, which are essential for these athletes to reach high performances. We looked for Electroencephalography (EEG) markers of CVSA usable for virtual reality-based NF training procedures, i.e., markers that comply with the following criteria: (1) specific to CVSA, (2) detectable in real-time and (3) related to goalkeepers’ performance/expertise. Our results revealed that the best-known EEG marker of CVSA—increased *α*-power ipsilateral to the attended hemi-field— was not usable since it did not comply with criteria 2 and 3. Nonetheless, we highlighted a significant positive correlation between athletes’ improvement in CVSA abilities and the increase of their *α*-power at rest. While the specificity of this marker remains to be demonstrated, it complied with both criteria 2 and 3. This result suggests that it may be possible to design innovative ecological training procedures for goalkeepers, for instance using a combination of NF and cognitive tasks performed in virtual reality.

## Introduction

In order to encourage sport practice and continue increasing peak performance of expert athletes, innovative training procedures that are both *motivating* and *efficient* should be proposed. So far, these innovative training procedures have mostly been dedicated to the improvement of physiological^1^ and biomechanical^2^ aspects of performance. Yet, sport performance is multifactorial. It is also shaped by neurophysiological, psychological, sociological and cognitive factors^3^, the influence of which has mostly been neglected. Taking these factors into account constitutes the next step of innovation in the understanding and improvement of sport performance. Indeed, their modulations could for instance explain why two players with the same physical abilities perform differently. We aim to contribute to this next step by designing innovative training procedures dedicated to the enhancement of athletes’ cognitive abilities. To do so, two main challenges should be taken on.

The first challenge concerns the acquisition of data that reflect specifically the target cognitive ability to be improved. Indeed, recording cognitive-related data (e.g., markers of attention) is less straightforward than recording biomechanical data (e.g., movements using motion capture systems) or physiological data (e.g., heart rate using an electrocardiogram). While the use of a cognitive ability may be associated with fluctuations of some physiological parameters such as the heart rate^4,5^ or the galvanic skin response^6^, these physiological responses are likely not to be specific to the target cognitive ability. They may be modulated by extrinsic factors (e.g., modulations of the room temperature) or intrinsic factors (e.g., circadian rhythm)^7^ independent from —or at least no fully related to— the task. Therefore, a more reliable indicator of the on-going cognitive process is likely to be the measure of neurophysiological patterns that specifically underlie this target cognitive process. Measuring such neurophysiological patterns requires the use of a brain signal recording method. In case of sport training procedures, the ElectroEncephaloGraphy (EEG), which is relatively cheap, transportable, and has a very good temporal resolution seems to be the most relevant and reliable tool available so far.

The second challenge to be taken on in order to design efficient cognitive training protocols concerns the feedback. Indeed, modulations of physiological and biomechanical parameters can be, to some extent, perceived by the athletes. They are associated with sensory and/or proprioceptive feedback. For instance, they can feel their heart beat increasing during a race. However, cognitive activities will mostly be associated with modulations of brain activity upon which athletes have no feedback. Yet, being provided with a feedback is a central and essential aspect of any training procedure. In order to address the absence of feedback *a priori*, one solution would consist in using a paradigm that enables athletes to be provided with a real-time feedback about their brain activity. Using this feedback, they could train to self-regulate the neurophysiological patterns underlying the cognitive ability of interest, and consequently improve this cognitive ability and potentially their sport performance. Such a paradigm is called a NeuroFeedback (NF) training procedure. Previous studies showing successful use of NF suggest that this approach can be adopted to train subjects to voluntarily self-regulate the activity of specific neurophysiological patterns, possibly entailing improvements in the cognitive capabilities underlain by these patterns.

In a typical NF training procedure, users are equipped with an EEG cap used to measure their brain activity, and more specifically the electrical currents generated by populations of cortical neurons. The EEG signal is pre-processed (e.g. using spatial and temporal filters) to enhance the neurophysiological features underlying the cognitive processes of interest. Based on these features, the system provides the subject with a feedback. This feedback can be a direct mapping of the features (standard NF approach) or it can be computed using a multivariate analysis approach (commonly adopted in standard Brain-Computer Interfaces –BCIs–). Using this feedback, one should learn to self-regulate the neurophysiological pattern underlying the target cognitive process, and thus control the feedback behaviour (e.g., to make it reach a predetermined threshold). A well-known use case of NF is Attention Deficit with-or-without Hyperactivity Disorder (ADHD) therapy^8,9^. In this case, NF is used to teach patients to self-regulate specific brain patterns (e.g., sensory-motor rhythms, *α* waves or *θ*/*β* ratio) in order to reduce the clinical symptoms of ADHD (e.g., to be able to focus their attention at school).

The first study in which NF efficiency has been tested to improve performance in a sport context was led in 1991 by Landers *et al*.^10^. Three groups of archers were included in the study, among which 1 passive-control group (no NF), one active-control group (NF with “incorrect“, i.e., uncorrelated feedback) and one experimental group (following a NF procedure based on the self-regulation of slow potential shifts). Participants of the experimental group significantly increased their performance (i.e., bow shots) from pre- to post-test while the active-control group showed a decrease in performance and the passive-control group showed no difference in performance between pre- and post-test. Nonetheless, the NF procedure was not associated with any clear modifications of neurophysiological patterns. Following this study, several other NF experiments have been led in the aim of enhancing athletes’ performance in different sports including golf^11,12^, swimming^13^, dance^14,15^ or athletics^16^. Mirifar *et al*.^3^ provided a systematic review of these NF studies. They included 14 studies, among which six investigated the self-regulation of Sensori-Motor Rhythms (SMR) (12–15 Hz on C3–C4 or Cz), two investigated the self-regulation of alpha-power over the sensori-motor cortex, two investigated the self regulation of the *α*/*θ* ratio over Pz, one investigated the self regulation of *α* and *θ* power over frontal areas and one investigated the self-regulation of slow cortical potentials over temporal areas. It has been concluded that, so far, a majority of published studies support the effectiveness of NF to improve athletes’ performance. Nonetheless, the specificity of the NF effect remains to be demonstrated. Indeed, it happened that the same protocol had different effects within the same or similar task, while it also happened that different protocols resulted in similar effects within a sport. Mirifar *et al*.^3^ stressed the fact that the quality of the studies included in the review was not always satisfying. Similar conclusions have been drawn by Xiang *et al*.^17^ following their meta-analysis of randomized controlled trials dedicated to the assessment of the efficiency of NF procedures to improve sport performance. This meta-analysis indeed revealed a significant effect of NF on both athletes’ sport performance and EEG self-regulation abilities. Nonetheless, it appeared that this effect depended on the control group design. In other words, the effect of NF on sport performance was not significantly different in experimental groups compared to active-control/placebo groups in well-controlled experiments, but it was significantly different from most of passive-control groups. The authors suggest that further studies, with better designed and organized trials, should be performed. Besides, as outlined by Park *et al*.^18^ the mismatch between lab studies conditions (both in terms of tasks, feedback and training environment) and real sporting conditions may explain, at least in part, the relatively little impact NF had on professional athletes and on their training routines so far.

In brief, by mainly targeting sensorimotor rhythms (which underlie movement representation, preparation and execution), the above-mentioned studies aimed at improving athletes’ performances through the improvement of their motor skills. Our project, however, aims at designing NF training procedures dedicated to enhancing athletes’ performance through the improvement of their cognitive skills. This project was initiated in the context of a collaboration between our research teams and the goalkeepers’ training staff of a French national first league soccer club. We decided to target a cognitive ability of the utmost importance for soccer goalkeepers, namely the Covert Visuo-Spatial Attention (CVSA). CVSA has been defined as the ability of committing attention to a position located in our peripheral field of view without any overt eye movement^19^. Having high CVSA abilities is essential for soccer goalkeepers who have to track the movements and positions of the players on the field (and most importantly of the player operating as the second forward) at the same time they follow the ball-holder. Importantly, since covert shifts of attention do not generate any observable behaviour, EEG provides access to a process that cannot be measured through any other modality. Moreover, although its efficiency is still being debated, NF has already been proven efficient to increase attentional abilities in the general population^20^. We want here to investigate the extent to which it could enable the improvement of CVSA abilities in soccer goalkeepers.

Covert attention has been shown to be underlain by specific neurophysiological patterns, and notably by a lateralised modulation of *α*-power (8–14 Hz) in the visual cortex^21,22^. More specifically, EEG studies have suggested that CVSA would be underlain by an increased power in the *α* frequency band over occipital areas (i.e., supposedly in the region of the visual cortex) ipsilateral to the hemi-field containing the object the person is focusing on. This increase would reflect an inhibitory process of the information coming from the controlateral hemi-field, thus allowing the allocation of more resources to the hemi-field of interest. Schmidt *et al*.^23^ have demonstrated in an EEG study that covert attention shifts to different target locations yielded distinctive topographical distributions of posterior *α* activity, paving the way for a NF paradigm. Consequently, the neurophysiological patterns underlying CVSA have been explored via the modulation of both the brain responses elicited by external visual stimuli^24^, and via the self-generated modulation of the activity in the visual cortex in a fully endogenous way (i.e., not depending on external stimuli)^25,26^. These covert shifts of attention have also been decoded in environments containing ambiguous spatial information^27^. Tonin and colleagues^26,28^ have demonstrated the feasibility of detecting covert shifts of visual spatial attention in real-time by analysing *α* sub-band modulations, building the foundations for using these neurophysiological patterns for real-time NF training procedures. Besides, Okazaki and colleagues^29^ have already succeeded in designing an MEG-based NF procedure that targeted posterior *α*. They have shown that this NF training led to alterations of *α* lateralisation and enabled an improvement of visual detection performance. Their results also suggested that a NF training might be detrimental for the performance of the untrained hemi-field. These encouraging results are also supported by an fMRI-based study which resulted in around 80% of accuracy for the recognition of 4 different target locations on a single-trial basis, using a single image volume^30^. While very insightful and encouraging, it should be noted that the possibility of measuring or training CVSA using fMRI or MEG-based NF does unfortunately not necessarily mean that it is possible to do so using EEG. Indeed, while being portable and affordable, and therefore more relevant for designing accessible sport training procedures, EEG also suffers from several limitations when compared to fMRI or MEG, including a low spatial resolution.

Thus, the previously mentioned literature enabled us to define our long-term objective, namely designing an innovative NF training procedure to improve soccer goalkeepers’ CVSA abilities and consequently their performance on the field. Based on this objective, the study depicted herein-after aimed to:

1. **Replicate the CVSA neurophysiological marker depicted in the literature -** Can we replicate the results from the literature, i.e. the lateralised modulation of *α*-power over occipital areas during CVSA tasks, in a population of soccer goalkeepers?
2. **Assess the relevance of the CVSA neurophysiological marker for a NF procedure -** In order for a neurophysiological pattern to be relevant for a NF procedure, it should comply with three main criteria: (1) it should be specific to the target ability (here to CVSA), (2) it should be detectable on a single-trial basis and (3) it should be related to athletes’ expertise and/or performance. Does the CVSA neurophysiological marker described in the literature comply with these criteria?
3. **Uncover alternative/additional and reliable CVSA neurophysiological markers -** Is the lateralised *α* modulation the only marker of CVSA? Or are there other neurophysiological markers that would be additional, perhaps more reliable and relevant to be used in a context of NF? These alternative markers reflecting CVSA abilities could either be generated during the covert attention task or be measured during resting periods.

**Figure 1:**
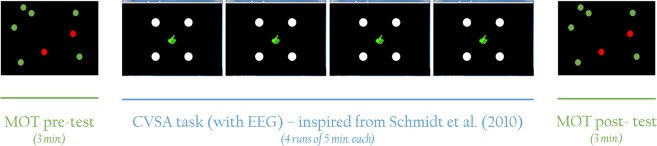
Scheme representing the development of one session, both sessions having the same structure. Participants were asked to complete the MOT test first for 3 min. Then, they performed 4 runs of the CVSA task (for around 25 min, breaks included) before completing the MOT test again at the end of the session.

In order to investigate these research questions, 17 volunteer goalkeepers were recruited for two experimental sessions that took place in the same week. Both sessions had the same structure. The goalkeepers performed a CVSA task based on the protocol of Schmidt *et al*.^23^, during which their EEG activity was recorded. They also completed a Multiple Object Tracking (MOT) test at the beginning and end of each session. The latter was used as a pre- and post-test in order to assess goalkeepers’ CVSA abilities, as well as the evolution of these abilities following the CVSA task (see Fig. 1). Indeed, beyond the fact that professional soccer goalkeepers use this test in their daily training to assess their covert attention abilities, it seems, based on the literature, that this test is quite appropriate for our purpose. First, Faubert *et al*.^31^ have shown that MOT performance was related to the athletes’ level of expertise. Second, it has been suggested by Parsons *et al*.^32^ and Kulpa *et al*.^33^ that MOT training procedures enable the improvement of attention abilities in healthy control adults, and of visuospatial attention abilities in soccer goalkeepers, respectively. Finally, in their study, Romeas *et al*.^34^, have demonstrated that a MOT training procedure enabled the improvement of soccer players’ performance on the field. This performance was measured in terms of passing decision making though. It cannot be claimed that this improvement was specifically due to an augmented efficiency of the information seeking strategy, itself enabled by enhanced CVSA abilities following the MOT training procedure. Nonetheless, the fact that MOT training procedures enable the improvement of performance on the field, together with the fact that they have been suggested to enable the improvement of attention abilities, suggest that the improvement on the field may be related to the improvement of CVSA, and that a MOT task is relevant to be used to assess this improvement.

## Results

### Descriptive analyses

One participant (S17) had to be rejected from the analyses due to EEG recording issues. The analyses were thus performed on 16 participants. Moreover, the EEG data of the first session of subjects 1, 2, 14 and 15, as well as the EEG data of the second session of subject 8 were not considered for the analyses due either to issues with the ground electrode or significant artifact contamination (see Table 1).

**Table 1:** Percentage of CVSA trials that were rejected due to gaze deviation, incorrectness of the behavioural response or EEG artefacts, for each participant.

Subjects’ IDs	% of Rejected Trials	
01	55.13%	*- with 4 fully rejected runs (i.e., 50.00%)*
02	52.29%	*- with 4 fully rejected runs (i.e., 50.00%)*
03	00.85%	
04	20.20%	*- with 1 fully rejected run (i.e., 12.50%)*
05	20.93%	
06	09.19%	
07	08.91%	
08	51.36%	*- with 4 fully rejected runs (i.e., 50.00%)*
09	06.61%	
10	00.85%	
11	05.57%	
12	33.65%	
13	02.13%	
14	51.25%	*- with 4 fully rejected runs (i.e., 50.00%)*
15	55.09%	*- with 4 fully rejected runs (i.e., 50.00%)*
16	06.69%	
17	100.00%	*- with 8 fully rejected runs (i.e., 100.00%)*

**Figure 2:**
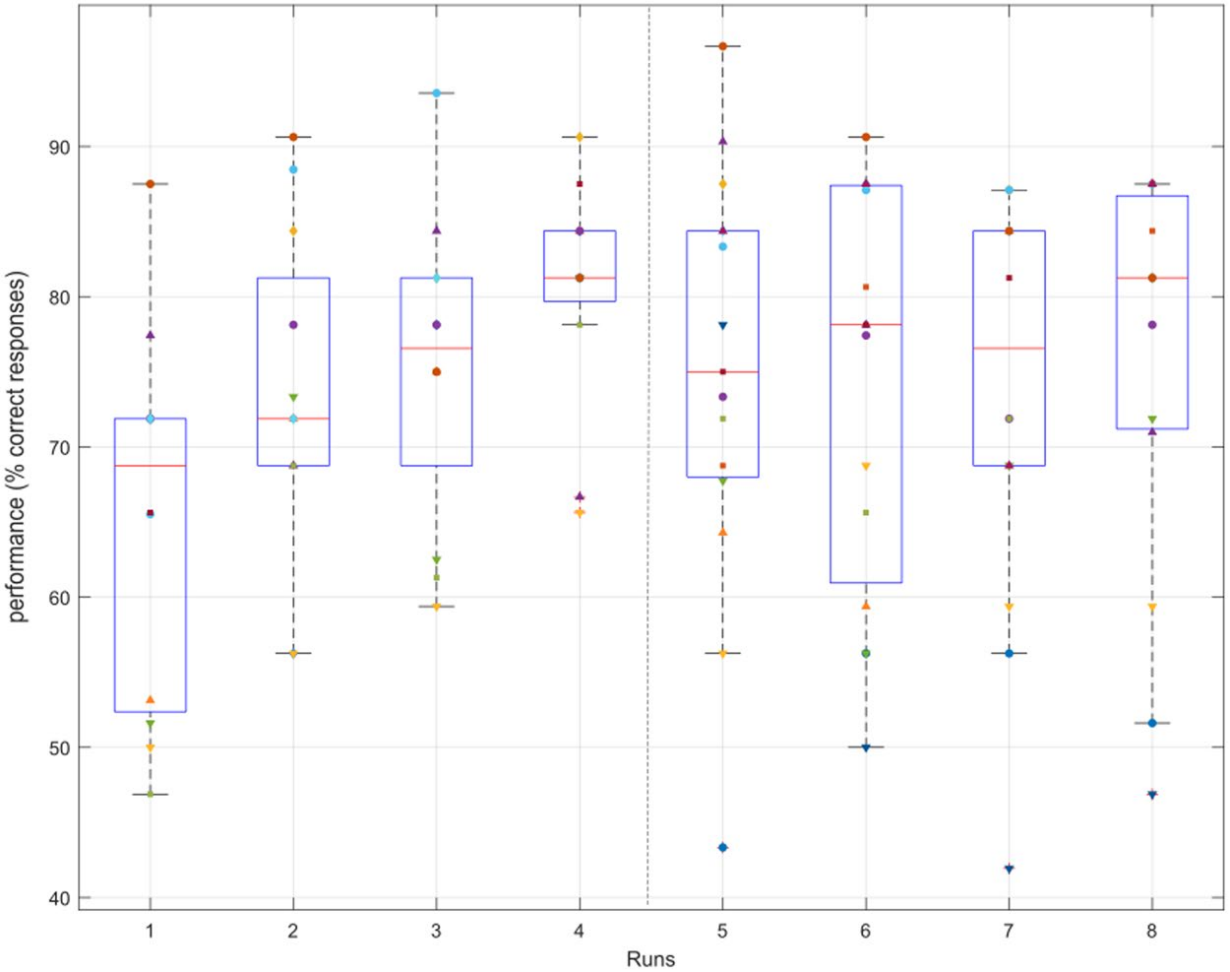
Boxplot representing the evolution of participants’ performances at the CVSA task over the runs (session 1: runs 1 to 4; session 2: runs 5 to 8), each point represents one participant.

**Figure 3:**
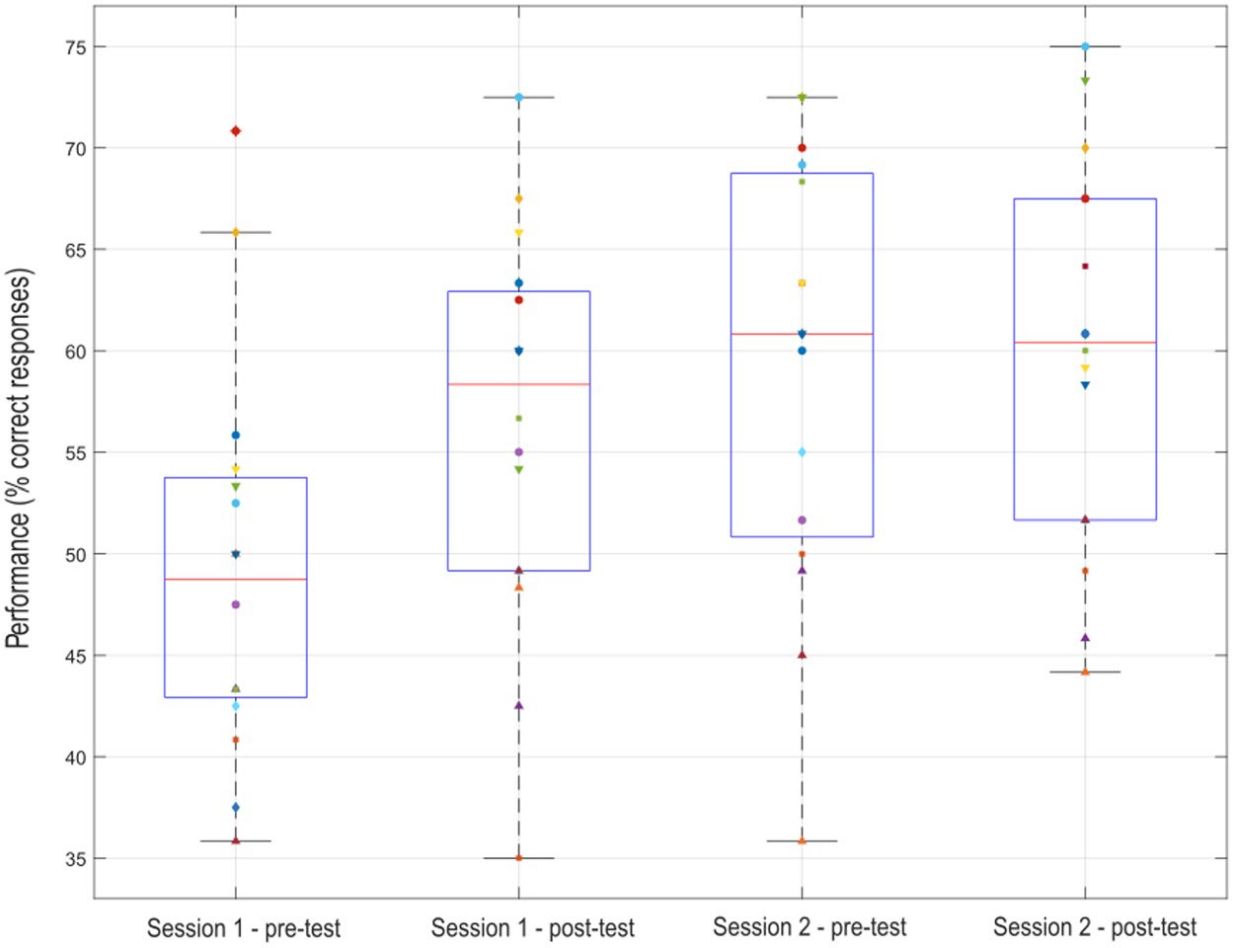
Boxplot representing the evolution of participants’ performances at the MOT task, each point represents one participant.

The CVSA task consisted for the participants in perceiving a sign (‘+’ or ‘×’) displayed in a target located in their peripheral field of view (up-left, up-right, down-left or down-right of the screen), and then in pressing a button accordingly. Goalkeepers obtained a mean behavioural performance (i.e., an average rate of correct button press) of 73.70% ± 7.81% during the first session, and of 74.40% ± 11.93% during the second session. As shown in Fig. 2, mean performances increased during the first session and then stagnated along the second session. Furthermore, the MOT test consisted in tracking 2 or 3 balls randomly moving on screen while focusing the gaze on a fixation point located at the center. MOT performances, measured as the average percentage of balls correctly tracked at each trial, were assessed in pre- and post-test for each session. Participants obtained the following MOT performances (see Fig. 3): ${\bar{X}}_{session1-pre}$ = 49.43% ± 9.48%, ${\bar{X}}_{session1-post}$ = 56.35% ± 9.74%, ${\bar{X}}_{session2-pre}$ = 59.22% ± 10.65%, ${\bar{X}}_{session2-post}$ = 59.95% ± 9.47%. These performances, and their evolution along the sessions, were associated with a high between-subject variability, with within-session evolutions ($MOTscor{e}_{post}$ − $MOTscor{e}_{pre}$) varying from −8.33% to 22.50% ($\bar{X}$ = 3.83% ± 7.33%) and between-session evolutions ($MeanMOTscor{e}_{Session2}$ − $MeanMOTscor{e}_{Session1}$) ranging from −9.17% to 19.17% ($\bar{X}$ = 6.25% ± 6.58%).

**Figure 4:**
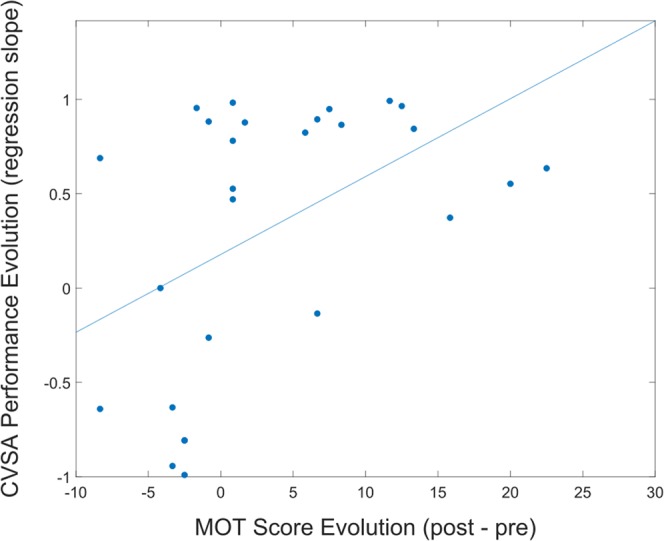
Graph representing the correlation between MOT score evolution and CVSA performance evolution during a session [Spearman correlation test - r = 0.5169, p ≤ 0.01]. This graph includes 2 points per participant, i.e., one per session.

Within-session MOT score evolution appeared to correlate with the within-session evolution of CVSA behavioual performance [Spearman correlation test - r = 0.5169, p ≤ 0.01], see Fig. 4. In other words, the more participants improved at the CVSA task along the session, the more they improved at the MOT test (post- vs. pre-test). This result suggests that the CVSA task may potentially be an efficient task for training this cognitive ability. Nonetheless, this hypothesis should be formally tested, for instance by comparing the MOT improvement pre- vs. post- CVSA task to the MOT improvement following a control task.

Furthermore, correlation analyses using Spearman tests revealed no significant correlation between the participants’ level of expertise and their CVSA abilities, measured through the performance they obtained at the MOT test and at the CVSA task. This absence of correlation may be due, at least in part, to the expertise measure that might not be reliable enough. Notably, the level at which athletes play does not always reflect their own level; also, the relationship between the level in the championship and the expertise required to play at this level is likely not to be linear. Therefore, more relevant metrics of expertise should be explored in the future.

### Replicating the CVSA neurophysiological marker depicted in the literature

**Figure 5:**
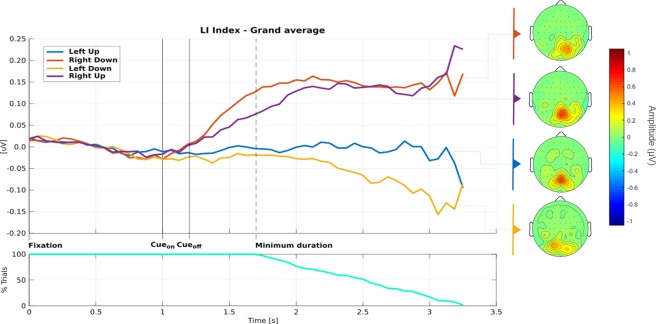
[TOP] Graph representing the LI as a function of the class, i.e., of the target location (blue: left-up, red: right-down, yellow: left-down, purple: right-up), and as a function of time. At t = 0 s, the fixation point is displayed. At t = 1 s, the cue (i.e., the instruction) is displayed for 200 ms. The signs (‘+’ or ‘×’) appear between 500 ms and 2000 ms after the cue disappears. [DOWN] Percentage of trials remaining for the computation of the LI. [RIGHT] Topography plots (averaged over all the trials of all the participants) computed over the last 500 ms of the trial. From top to bottom: right-down, right-up, left-up, left-down.

We used the Lateralisation Index (LI) as a measure of the phenomenon of lateralisation of the *α*-power during the CVSA tasks. The LI was computed as the difference of *α*-power between the right and left hemispheres, above the parieto-occipital areas (more details about the LI computation are provided in the Methods section). Group analyses were performed in order to assess the replicability of the LI marker. Figure 5 (top) shows the average LI over participants as a function of the target location and as a function of time. Figure 5 (down) shows the number of trials on which the LI was computed. Indeed, the sign (‘+’ or ‘×’) was randomly displayed between 500 ms and 2000 ms after the cue. Thus, as the time increased, the number of trials available for each time point decreased. Consequently, the variability of the reported grand-average LI increased. Consistently with previous studies^23,26^, the LI appeared to be positive when the target was located on the right, negative when it was located on the left. It reflects a higher *α*-power over the occipital cortex ipsilateral to the target.

Based on this first (consistent) result, we investigated the influence (1) of the class (i.e., of the location of the target: up-left, up-right, down-left, down-right) and (2) of the duration of the trial, on the LI. A 2-way ANOVA for repeated measures with the *Duration* (D_3_: short, medium, long) and the *Class* (C_4_: left-up, left-down, right-up, right-down) as independent variables and the LI as dependent variable was performed. This ANOVA revealed a strong main effect of the *Class* [F(3,45) = 8.090, p ≤ 0.001] as well as a tendency towards a main effect of the *Duration* [F(2,30) = 2.848, p = 0.079], but no interaction effect between those two factors [F(6,90) = 1.213, p = 0.311]. This analysis suggests that the LI differs significantly as a function of the target location. It also suggests that the longest the trial, the largest the LI. Finally, the absence of interaction between the *Class* and *Duration* indicates that the time required for the LI to develop is not related to the target location. Coupled with the analysis of the graph representing the LI (Fig. 5, left), the visual inspection of the topography plots (Fig. 5, right) seems to indicate a stronger lateralisation when targets are located in the lower part of the screen, which is in line with previous findings in literature^35,36^.

### Assessing the relevance of the CVSA neurophysiological marker for a NF procedure

**Figure 6:**
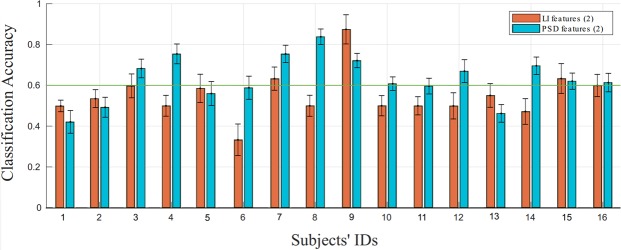
Classification Accuracy (CA) for each subject and each classifier, i.e., LI and PSD classifiers (that were both based on 2 features). The green horizontal line represents the chance level (60% of CA).

As indicated earlier, in order for a neurophysiological pattern to be relevant for a neurofeedback procedure, it should comply with three main principles. First, it should be specific to the ability to be trained, in order to ensure that the training procedure does target a specific process and that the reported improvement is not related to a general cognitive training procedure. Second, it should be detectable on a single-trial basis in order to enable us to provide athletes with ‘real-time’ feedback. Finally, at least one of its characteristics (e.g., its latency or amplitude) should correlate to a measure of performance/expertise reflecting the target ability, in order for the athletes to know how they should improve (e.g., should they learn to up-regulate or down-regulate the neurophysiological pattern). According to the literature and our results, the LI is specific to CVSA^21,22^ (it complies with the first criterion). The compliance of this marker with the last two criteria remained to be tested. In order to evaluate the extent to which the LI was detectable on a single-trial basis, we performed offline trial-based classification analyses. We decided to train and test the classifier on the discrimination of only two classes, namely the bottom-left and bottom-right classes, which were the ones eliciting the most different LIs (see Fig. 5). The classifier was based on two features characterising the LI: its amplitude (LIA) and its latency (LIL). The computation of the LIA and LIL features as well as the whole classification process are described in the Methods section. The average classification accuracy (CA) was around chance level^37^ ($\bar{X}$ = 55.11% ± 5.72%, *range*_*CA*_ = [33.33%; 87.50%], see Fig. 6). This LI-based classifier was able to discriminate bottom-left vs. bottom-right CVSA with performances higher than chance (which was set to 60%^37^) for only 4 athletes out of 16, and for these athletes, the average CA was of only 68.54%. Furthermore, in order to assess for the compliance of the LI with the third criterion (i.e., its relationship with performance/expertise), we performed correlation analyses. These analyses revealed no significant correlation between the LI (more precisely the absolute value of the LIA and the LIL) and the performance (CVSA and MOT score) or level of expertise (level in the French championship).

To summarize, while the LI had been shown to be specific to CVSA (first criterion), it appeared not to be sufficiently reliable for single-trial analyses (second criterion) and not directly related to performance/expertise measures (third criterion). Therefore, other approaches, based on machine learning techniques were used to determine whether or not other neurophysiological markers of CVSA could be defined. These approaches are depicted in the next section.

### Uncovering alternative/additional and reliable CVSA neurophysiological markers

In a first attempt to characterise alternative or additional neurophysiological markers of CVSA that would comply with the three criteria, we performed offline classification analyses based on a multivariate classification approach, that is described in the Methods section. The average classification accuracy (CA) of the obtained Power Spectral Density (PSD)-based classifier was above chance level [$\bar{X}$ = 63.03% ± 4.56%, *range*_*CA*_ = [42.12%; 83.81%] (see Fig. 6). This classifier was able to discriminate bottom-left vs. bottom-right target CVSA activity with performances higher than chance for 10 athletes out of 16. A Wilcoxon Rank sum test enabled us to show that the PSD-classifier was associated with significantly higher performances than the LI-based classifier [Z = 1.95, p ≤ 0.05]. Nonetheless, it should be noted that while the number of athletes for whom the classification accuracy was above chance level was more than twice higher with this second classifier, the average classification accuracy for these athletes was similar to those obtained using the LI-based classifier, i.e., 69.62% on average.

To summarise, the PSD classifier’s performances (in terms of classification accuracy) appeared to be rather low, although better than the LI classifier’s. Several levers could be used in the future in order to improve the performances of this PSD classifier, e.g., the selection of an individualized number of features for each user or the use of a more relevant decision making algorithm. Beyond its current modest reliability, this approach may be difficult to use in practice due to the high within- and between-subject variability, which prevents one from knowing *a priori* which patterns should be targeted during the NF procedure. Using this approach, we obtained markers that were specific to CVSA (i.e., complied with the first criterion) and detectable in single-trial with a–while rather low–better-than-chance performance (i.e., partially complied with the second criterion). Nonetheless, these markers, i.e., the features selected by the PSD classifier, did not correlate with the participants’ performances or expertise (i.e., did not comply with the third criterion).

**Figure 7:**
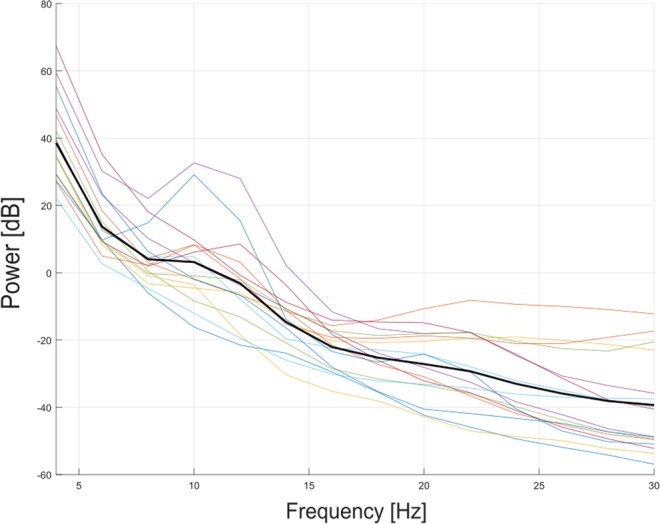
Graph representing the spectrum of pre-cue *α*-power for each participant. The bold line represents the spectrum averaged over all the participants.

**Figure 8:**
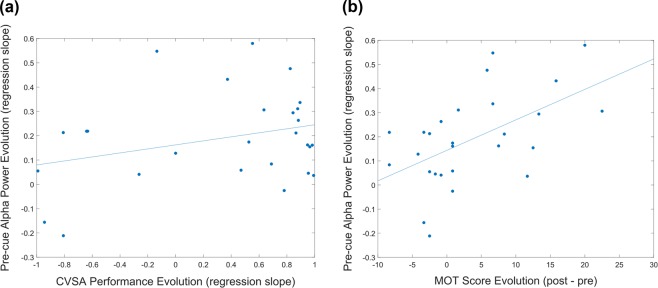
(**a**) Graph representing the correlation between CVSA performance evolution during a session and the evolution of the pre-cue *α*-power [Spearman correlation test-r = 0.1062, p = 0.5989]. **(b)** Graph representing the correlation between MOT score evolution and the evolution of the pre-cue *α*-power [Spearman correlation test - r = 0.4799, p ≤ 0.05]. Both these graphs include 2 points per participant, i.e., one per session.

Consequently, we decided to adopt a different approach. Rather than investigating the modifications of EEG activity during CVSA tasks, we looked for markers of covert attention abilities improvement in the EEG activity at rest. More precisely, we computed the average amplitude of the Individual Alpha Peak (IAP - see the Methods - CVSA classification Section) during the pre-cue resting period (for more details the computation of pre-cue *α*-power, please refer to the Methods section). The spectrum of pre-cue *α*-power for each participant is depicted in Fig. 7. Our analyses revealed no significant correlation between CVSA performance evolution and the evolution of pre-cue *α*-power [Spearman correlation test-r = 0.1062, p = 0.5989] (see Fig. 8a). However, a significant positive correlation was revealed between MOT score evolution and the evolution of pre-cue *α*-power [Spearman correlation test-r = 0.4799, p ≤ 0.05] (see Fig. 8a). In other words, the more the pre-cue *α*-power amplitude increased along the session, the more participants improved at the MOT test. Therefore, this last marker complies with the third criterion, its amplitude correlates with athletes’ performance. It also complies with the second criterion as the *α*-power can be detected in single trials. Nonetheless, its specificity to CVSA, i.e., its compliance with the first criterion, remains to be demonstrated.

## Discussion

The aim of this study was to investigate neurophysiological (EEG) correlates of Covert Visual Spatial Attention (CVSA) that could be used for NF training procedures dedicated to the improvement of soccer goalkeepers’ performances. We defined three criteria that a neurophysiological marker should fulfill in order to be relevant and usable for NF. These markers should be (1) specific to the target ability (here the CVSA), (2) detectable on a single-trial basis and (3) be characterized by at least one feature that correlates to athletes’ performance or expertise. The results of our experiment (N = 17 goalkeepers, who took part in 2 sessions each) revealed that the best-known neurophysiological correlate of CVSA was not suitable for NF. Indeed, this lateralized increase of *α*-power over the occipital cortex ipsilateral to the attended hemi-field fulfilled the first criterion (specificity) but not both the other criteria. It was not detectable on a single trial basis and did not correlate to the athletes’ performance or expertise. Consequently, we used a different approach, based on a multivariate analysis (BCI-oriented), and more precisely on power spectral density analyses, to determine neurophysiological patterns underpinning CVSA that could reliably be used for a NF procedure. This approach enabled us to highlight patterns specific to CVSA (i.e., complying with the first criterion) and detectable on a single-trial basis with performances above chance level (i.e., complying with the second criterion). Nonetheless, these patterns did not correlate to athletes’ performance/expertise (i.e., did not comply with the third criterion), and the their detection performance (around 63% of good recognition rate) was not satisfying enough. What is more, the patterns selected using this pure machine learning-based approach suffered from a high within-subject and between-subject variability. Therefore, we believe that this approach, in its current state, is not reliable enough for a NF procedure. While these PSD analysis performances could be improved, for instance by introducing a subject-specific feature selection or by using more adapted decision-making algorithms, this solution would require an entire study to be comprehensively explored. Hence, in the aim of uncovering alternative or additional EEG markers, we investigated the extent to which improvements in CVSA abilities might be underlain by modifications of the EEG activity at rest (by opposition of modifications of EEG activity during CVSA tasks). Our results revealed that the evolution of the *α*-power over occipital areas at rest during pre-trial periods positively correlated to the improvement of covert attention abilities (measured using MOT scores). In other words, the more their *α*-power at rest increased during one session, the more goalkeepers improved their MOT scores from pre- to post-test. This last marker thus complies with both criteria 2 and 3. Nonetheless, its specificity remains to be demonstrated.

Moreover, further analyses will be required in order to determine if this correlation actually reflects a causal effect. In other words, in a future experiment, we should determine if (1) the increase of *α*-power results from the CVSA training, if (2) it reflects a generic idling state that enables a better flexibility to adapt to the task –in which cases it would be the consequence of CVSA abilities’ improvement or a non specific effect induced by the cognitive training, respectively, and not a direct marker of these abilities–, or if (3) it is a neurophysiological adaptation that enables and underlies CVSA abilities’ improvement, in which case it would be a direct marker of these abilities. In order to disentangle these different hypotheses, one option could consist in assessing whether or not an increase of *α*-power at rest, e.g., enabled by a simple NF training procedure, would result in an improvement of CVSA abilities. If such was the case, innovative and promising NF procedures, during which soccer goalkeepers would be asked to learn to up-regulate their *α*-power over parieto-occipital areas, could be proposed to enable the improvement of these athletes’ performances. Even if the increase of *α*-power at rest were to be shown not specific to CVSA, a NF procedure would still be promising and in line with the literature as it has been demonstrated that having a large *α*-power “in a reference interval preceding a task [*was*] related to both large suppression of upper *α*-power during the task and good performance”^38,39^. Some studies^40,41^ have even shown that NF training procedures can be used to train subjects to up-regulate their *α*-power (and more specifically in these studies their upper-*α*), and consequently improve their performances at cognitive tasks (here, mental rotation tasks). Hanslmayr *et al*.^40^ have highlighted the fact that following a NF training procedure, NF responders (i.e., those who managed to up-regulate their *α*-power) “showed a significant increase in reference upper *α*-power (i.e., in a time interval preceding mental rotation)”. This knowledge, together with our results showing a significant correlation between the progression of covert attention abilities (measured using MOT scores) and both the evolution of pre-cue *α*-power and the evolution of performance at the CVSA task, suggest that a NF training procedure targeting parieto-occipital *α* could be used as a pre-training, or cognitive preparation, in order to improve CVSA abilities. Among different possibilities, we could for instance ask soccer goalkeepers first to follow a NF training procedure (up-regulation of *α*-power) as a general cognitive training, and then ask them to perform CVSA tasks to improve specifically their covert attention abilities. The NF training should indeed enable them to have higher *α*-power in pre-trial, which could potentially result in stronger *α* de-synchronisation, and thus higher performances during the task. Further investigations should then be led in order to determine the transferrability of these improvements to performances on the field. While promising, it should be noted that, currently, NF training procedures suffer from a low level of efficacy that is notably related to the high percentage of “non-responders”. This issue is currently being investigating. Notably, a lot of effort is dedicated to better understand the factors (e.g., users’ states and profile) influencing one’s responsiveness to NF in order then to design procedures adapted to each user/athlete^42^. Finally, beyond this aspect of NF efficacy, an exciting challenge (in order to achieve high proficiency) will consist in integrating this procedure in a coherent and motivating global training strategy which enables an efficient transfer of skills on the field, possibly using virtual reality.

## Methods

### Participants

Seventeen EEG-naive soccer goalkeepers (2 women, 15 men; aged 21.4 ± 5.3 year-old) took part in this 2 session-long experiment. All participants but two were right-handed. They all completed and signed an informed consent form for study participation at the beginning of the first session. Moreover, due to their status, professional goalkeepers were informed that the collected and published data may enable their identification. Consequently, all the professional athletes who took part in the experiment completed and signed an informed consent stating that they agreed for the publication of identifying information/images in an online open-access publication. The experimental procedure was led in accordance with the Declaration of Helsinki and was approved by the Inria Ethics Committee (reference: 2017-021/01).

### Experimental procedure

**Figure 9:**
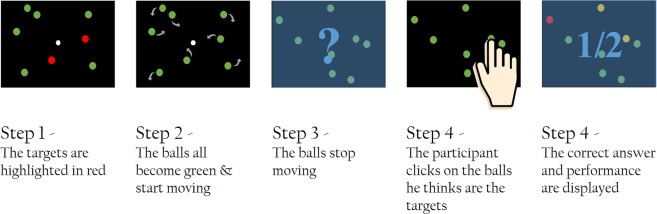
Scheme representing the different steps of a MOT trial. First, the balls to be tracked are highlighted in red. When the participant is ready, they press the space bar and the balls start moving at the same time they all become green. The participant should track the balls of interest while keeping their gaze on the fixation point (the size of which has been increased to be visible on this scheme). A few seconds later, the balls stop moving and the participant is asked to click on the balls they think are the balls to be tracked. They are provided with a feedback indicating the correct balls as well as their performance. When they are ready, they can run the next trial.

**Figure 10:**
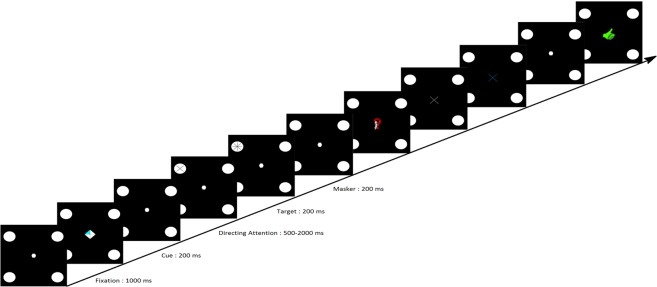
Scheme representing the timing of a trial during the CVSA task (inspired from Schmidt *et al*.^23^). A trial starts with a fixation period of 1000 ms. The participant is asked to maintain their gaze on the fixation point located at the center of the screen for the whole trial (the size of the fixation point has been increased in the Figure in order to be visible). Then, the cue (diamond with one quarter highlighted in blue) is displayed for 200 ms. Between 500 ms and 2000 ms after the cue disappeared, a sign (‘+’ or ‘×’) is displayed in the indicated target for 200 ms. The sign is immediately followed by a marker for 200 ms. Then, the participant is asked to click on the button representing the sign they perceived. Once their choice was validated, they are provided with a feedback indicating if their response was correct of not. Each CVSA run includes 32 trials.

All participants took part in two sessions of approximately one hour each. Both sessions took place in the same week. At the beginning of the first session, participants filled and signed the consent form as well as a general information questionnaire containing questions about their age, gender, and number of hours of sleep. Then, both sessions had the same structure, see Fig. 1. Participants performed 4 runs (sequences) of a CVSA task during which their EEG activity was recorded. They also completed a MOT test at the beginning and at the end of each session. Thus, both sessions were divided into 3 parts: the MOT pre-test (that lasted 3 min), followed by the CVSA task during which the participants’ brain activity was measured (for around 25 min), and the MOT post-test, again for 3 min. The MOT pre- and post-tests were divided into 20 trials of around 10 s each. These trials consisted in tracking 2 or 3 balls moving on screen among 7/8 or 11/12 other balls, respectively, while fixating a point at the centre of the screen. At the beginning of each trial, the balls to be tracked were highlighted in red, while the others were green coloured. When ready, the participants pressed “space bar” on the keyboard. From that moment, all the balls started moving and the balls to be tracked became green (just as the other balls). A gaze tracker was used in order to check that the participants maintained their gaze on the fixation point while tracking the target balls. If ever their gaze deviated, they would hear a beep. The trials containing a gaze deviation were considered as “failed”. At the end of each trial, the participants had to click on the target balls that they had been asked to track. They were then provided with a feedback indicating the correctness of their response. After performing this test a first time (for around 3 min), participants were equipped with an EEG cap and performed the CVSA task that was divided into four sequences of around 5 min each. Each sequence (or run) was itself divided into 32 trials. At the beginning of each trial, a fixation point was displayed at the centre of the screen. Here again, participants were asked to focus their gaze on this point and not to deviate until the end of the trial. A beep sound was also issued if ever their gaze deviated, which was associated with a “fail” for the on-going trial and the rejection of the trial from the EEG analyses. At the same time the fixation point appeared, 4 round targets were displayed at the four corners of the screen. One second later, a white diamond divided into four quarters appeared for 200 ms in the centre of the screen. One quarter of this diamond was highlighted in blue, which corresponded to the location of the target to which the participant was instructed to focus their attention on. For instance, if the top-left quarter of the diamond was highlighted, the participant was supposed to focus their attention on the top-left round target while still maintaining their gaze on the fixation point located at the centre of the screen. Between 500 ms and 2000 ms later, either a ‘+’ or a ‘×’ sign was displayed inside the target for 200 ms, immediately followed by a masker (which was a star, i.e., a combination of both the ‘+’ ad ‘×’ signs) also displayed for 200 ms. These signs and maskers were always displayed on the target on which the participants had been instructed to focus. In other words, no sign was displayed on unattended targets. Following the disappearance of the masker, an interrogation point indicated that the participant should select, using a pad, which sign they had perceived. To do so, they had to click a first time on the button corresponding to the sign they had perceived. This sign was then displayed in white on screen. Consequently, they could either change their mind and click on the other button (corresponding to the other sign) or click a second time on the same button to validate their choice. The validation resulted in the sign on screen turning blue. Immediately following their validation, participants were provided with a feedback: a green thumb up or a red thumb down depending on whether their response was correct of incorrect, respectively. Finally, a break lasting between 1000 and 3000 ms preceded the next trial. As mentioned previously, there were 32 trials per run (or sequence), i.e., 8 for each target, and 4 runs in total (each run lasting around 5 min). The MOT test enabled us on the one hand to assess intra-session improvements following the covert attention task, and on the other hand to have a standard, validated measure, to compare athletes’ CVSA abilities. An illustration of the MOT interface is provided in Fig. 9 while the covert attention task (interface and timing) is described in Fig. 10.

### Experimental setup

**Figure 11:**
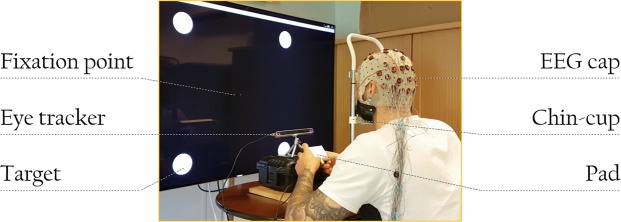
Photo of a professional soccer goalkeeper taking part in the CVSA task.

During the experiment, participants were seated in front of a table on an office chair adjustable in height, see Fig. 11. Their head was placed on an adjustable chin-cup, so that their eyes were on the same horizontal plane as the centre of the screen, where the fixation point was displayed. The chin-cup was used in order to increase participants’ comfort, and help them to maintain their position and distance from the screen during the experiment. It also facilitated the gaze recognition by the eye tracker (we used an Eye Tribe - http://theeyetribe.com/). A gaze deviation of 1.5° of visual angle from the fixation point was tolerated due to the “limited” precision of the Eye Tribe (0.5° of visual angle). The Eye Tribe was placed in front of the participant, at a distance of 40 cm. It provided gaze direction information in near real-time (latency of 20 ms) and was used in a 60 Hz sampling rate mode. Participants were placed at 70 cm of a 55 inch television screen. The four target circles had a size of 6.1° of visual angle and they were presented at an eccentricity of 27° from the fixation point, in order to ensure that they were located in the participants’ peripheral field of view, which is the “vision produced by light falling on areas of the retina outside the macula”^43^, and that they were displayed inside and close to the limit of 30° retinal eccentricity, which is the limit from which the visual acuity decreases more strongly^44^. The MOT exercise was implemented in Unity 5.0 using C#. The Covert Attention task was implemented in C++ as an OpenVibe scenario^45^. This set-up was hold by a laptop equipped with an I7 Core processor and 32 Gb RAM.

### Recording and preprocessing of the EEG Data

EEG data was recorded using two g.USBAmp amplifiers (g.tec, Austria), by means of 32 wet (g.tec LadyBird) scalp electrodes (F3, F1, F2, F4, FC3, FCz, FC4, C1, Cz, C2, CP5, CP3, CPz, CP4, CP6, P7, P5, P3, P1, Pz, P2, P4, P6, P8, PO7, PO3, POz, PO4, PO8, O1, Oz, O2, 10–20 system), referenced to the right ear and grounded to AFz (see Fig. 11). Such electrodes cover the whole scalp, with a higher density above the occipital cortex, which is our main area of interest based on the literature. Indeed, as previously mentioned, covert attention would be underlain by a lateralized modulation of the brain activity in the *α* band (8–14 Hz) over visual areas (occipital cortex). EEG data were sampled at 512 Hz. For the offline analyses, EEG signals were band-pass filtered in 1–40 Hz and epoched. The epochs started when the cue (i.e., diamond) was displayed at the centre of the screen, and ended when the symbol (i.e., ‘+’ or ‘×’) was displayed in the round target. Then, a semi-automatic method was used for artifacts removal: for each channel, epochs for which the mean amplitude of the signal was (1) higher than 5 standard deviations of the average signal amplitude across the trial and (2) larger than 80 *μ* V were marked down and rejected after visual inspection.

Then, data were re-referenced using Common Average Referencing (CAR). Finally, a baseline removal algorithm was applied. To do so, for each run, the average power of the signal at rest, in pre-trial (i.e., 0.250–0.750 s after the fixation point) was computed and then subtracted from the in-trial signal power. In addition, trials associated with a gaze deviation were rejected. The data were recorded using the OpenViBE software^45^ and pre-processed using Matlab/EEGLab^46^.

### Behavioural and neurophysiological analyses

#### Behavioural analyses: CVSA performance & expertise

Three different metrics were used in order to assess the level of CVSA performance and the expertise of the participants. First, regarding the CVSA performance, both the performances obtained at the MOT and CVSA tasks were considered. MOT performances were measured as the average percentage of balls correctly tracked at each trial. They were assessed in pre- and post-test for each session. On the other hand, CVSA performances were computed as the average rate of correct button presses for each run along the training. Finally, the expertise was approximated through the allocation, to each participant, of a score corresponding to the level at which they played in the French championship (e.g., 1 = fifth level of a district championship; 8 = first level of a regional championship; 13 = french first league; 14 = national team), see Table 2. Then, 3 non-parametric Spearman correlation analyses have been made in order to assess the relationship between these metrics of performance and expertise. More specifically, these correlation analyses were computed between: (1) the intra-session evolution of MOT performances (computed as the difference between post and pre-test performance for each session), (2) the intra-session CVSA performance evolution (computed as the regression slope calculated from the CVSA performance obtained at each run) and (3) the expertise score. It should be noted that given the small number of comparisons made (12 in total in the paper), no correction for multiple comparisons was performed.

**Table 2:** Number of athletes for each expertise level.

Level ID	Name of the Level in the Championship	Number of Athletes
14	National Team	2
13	Ligue 1	3
12	Ligue 2	—
11	National 1	2
10	National 2	2
9	National 3	2
8	Regional 1	1
7	Regional 2	1
6	Regional 3	1
5	District 1	1
4	District 2	1
3	District 3	1

#### EEG analyses: lateralisation index (LI)

The LI was computed as the difference of *α*-power between the right and left hemispheres, above the parieto-occipital areas. For more rigorous analyses, we used participant-specific *α* frequency ranges. These individual *α* frequency bands were computed as follows. We first computed the frequency and the amplitude of the individual alpha peak(s) (IAP) for all the pre-trial periods, in the 6–16 Hz frequency band. If several IAPs were detected within one pre-trial, we selected the one with the largest amplitude. For each participant, the global IAP frequency used to compute the LI was the median of the IAP frequencies selected over the trials. The *α*-power band for the LI computation was set as the following interval: [IAP_*global*_ − 2; IAP_*global*_ + 2] Hz. Next, we identified two Regions of Interest (RoI), namely the Left (P7-P1, PO7, PO3, O1) and Right (P8-P2, PO8, PO4, O2) parieto-occipital areas. We computed the Lateralization Index (LI) as follows:

$$\begin{array}{c}Fortin\,{\rm{\text{[cue ; end-of-trial]}}}:\\ LI(t)=L{I}_{trial}(t)-\bar{L{I}_{baseline}}\\ -\\ with:\\ L{I}_{trial}(t)={\alpha {\text{-power}}}_{Right}(t)-{\alpha {\text{-power}}}_{Left}(t)\\ and:\\ Forbin\,[{\rm{\text{baseline-start}}};{\text{baseline-stop}}]:\\ L{I}_{baseline}(b)={\alpha {\text{-power}}}_{Right}(b)-{\alpha {\text{-power}}}_{Left}(b)\end{array}$$

Thus, for each trial, the LI was normalised through its subtraction by the average LI recorded during the pre-trial baseline. The baseline started 750 ms before the cue (“baseline-start”) and ended 250 ms before the cue (“baseline-stop”). This method of normalisation enabled us to highlight the absolute amplitude of the LI in order to investigate the between-subject variability, and more specifically the variability due to performance and expertise. It should be noted that more standard methods, consisting for instance to divide the difference by the sum of both terms (i.e., to highlight the relative amplitude), while relevant for within-subject analyses, may have prevented us from observing these between-subject variabilities.

Then, a 2-way ANOVA for repeated measures with the *Duration* of the trial (D_3_: short, medium, long) and the *Class* (C_4_: left-up, left-down, right-up, right-down) as independent variables, and the LI as dependent variable was performed in order to investigate the evolution of the LI along the trial as a function of the target location. The trials were divided into three categories, namely short, medium and long trials, as a function of the delay between the disappearance of the cue and the display of the sign in the target. In short trials, the sign was displayed between 500 ms and 1000 ms after the cue disappeared. In medium trials, it was displayed between 1000 ms and 1500 ms while in long trials, it was displayed between 1500 ms and 2000 ms after the cue disappeared. For all durations, we used the last 500 ms of the trial for the analyses.

Finally, 6 non-parametric Spearman correlation analyses were performed in order to assess the relationship between the metrics of performance and expertise described herein above and the LI. To do so, we computed two features illustrating LI characteristics, namely its amplitude (LIA) and its latency (LIL). The LIA was computed as the average value of the LI over the last 500 ms of the trial. In order to compute the LIL, the mean LI_*pre-trial*_ as well as its standard deviation were computed during the pre-trial period (resting period). Thereupon, the LIL was computed as the first time point (during the trial) at which the absolute value of the LI was higher than the mean LI_*pre-trial*_ plus two standard deviations.

#### EEG analyses: CVSA classification

**Figure 12:**
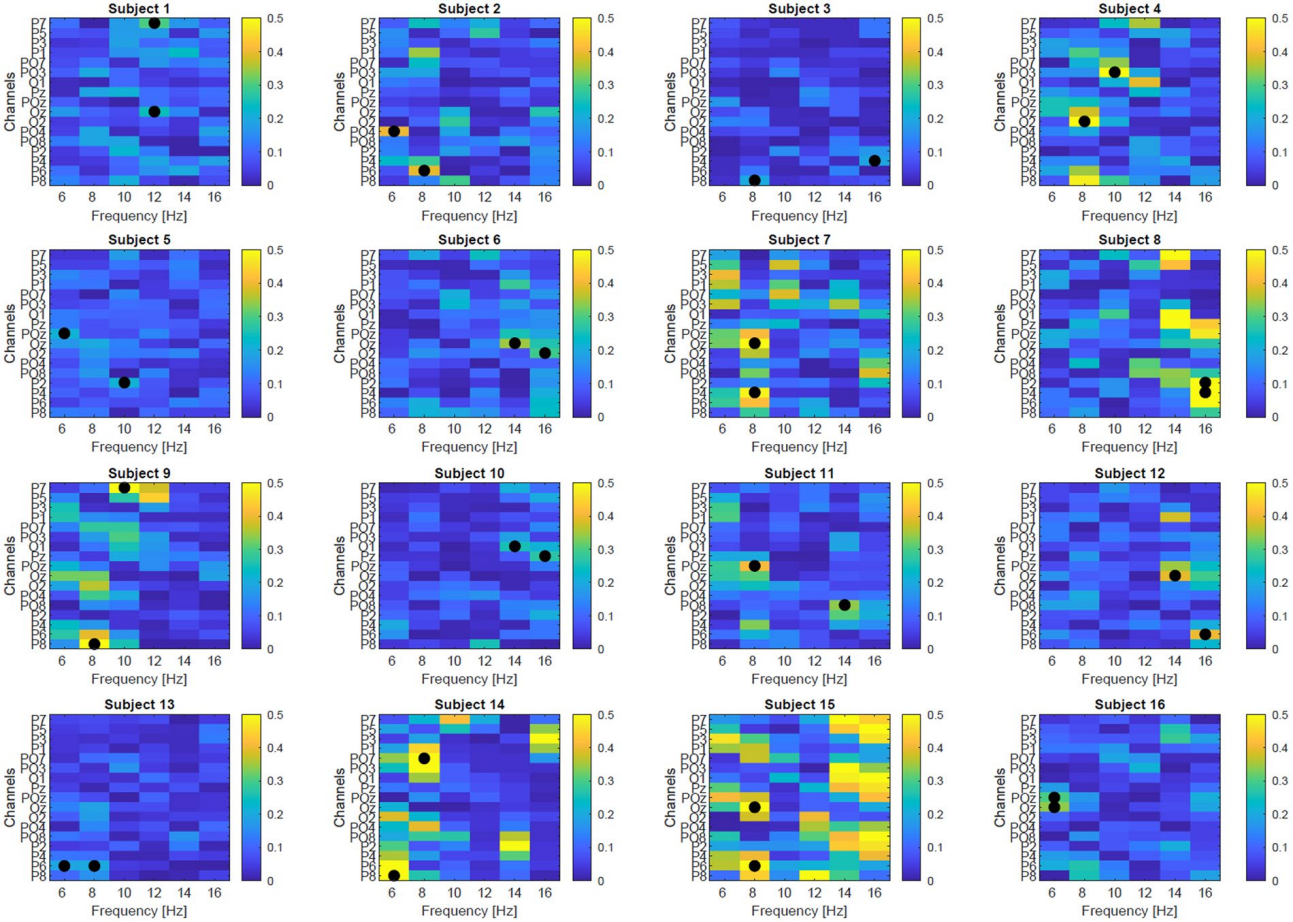
Feature discriminancy maps per subject computed on the 8 runs. Bright color (yellow) indicates high discriminancy between bottom-left and bottom-right CVSA task. The discriminancy of each feature (channel-frequency pair) is quantified as the Fisher score of the PSD for the two CVSA tasks. For each subject, the two most discriminant features are marked with black circles and they have been used as input for the PSD classifier.

Two different classifiers, called the LI classifier and the PSD classifier, have been designed in order to assess the possibility of discriminating between left and right covert attention in a single trial basis. These offline classifiers considered two classes, namely the bottom left and bottom right targets, which were the ones eliciting the most different LIs (see Fig. 5). The LI classifier was based on two features related to the LI: its amplitude (LIA) and its latency (LIL). For each session, the data of the first three runs was used to train a Gaussian decoder with a gradient-descent supervised learning approach^47,48^ and the data of the fourth run was used to test it. It should be noted that only the “correct trials”, i.e., those for which the participant provided the correct behavioural response (pressed the correct button) were included in the training and testing data sets. The PSD classifier on the other hand was based on a multivariate classification approach. Thus, we first computed the Power Spectral Density (PSD) of EEG signals via a Welch’s periodogram algorithm with 2 Hz resolution (from 4 to 48 Hz) in 1-second windows sliding every 62.5 ms. Second, we extracted the PSDs in the covert attention period and performed feature selection based on the Fisher score value of each feature (i.e., channel-frequency pair) in the parieto-occipital regions. The two most discriminant features were selected as input for the Gaussian decoder (see Fig. 12). We selected only the two best features in order for this classifier to be comparable to the LI-based classifier, which was also fed with two features (the LIA and LIL). Similarly to the LI-based classifier, the 3 first runs were used to train the classifier while the last one was used to test it, and only the correct trials were considered. This PSD-based classifier yielded to a sample-based classification. In order to obtain a trial-based classification (the LI classifier resulting in a trial-based classification), we applied a simple decision-making algorithm computing the majority vote.

#### EEG analyses: pre-cue *α*-power

**Figure 13:**
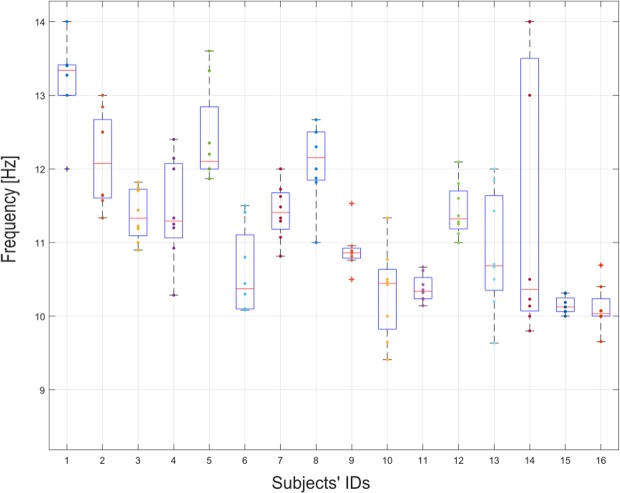
Boxplot representing the Individual Alpha Peak (IAP) distribution for each participant. Each point represents the average IAP during the pre-cue resting period for one run.

In order to investigate potential markers of CVSA during rest periods, we computed the average amplitude of the Individual Alpha Peak (IAP - see the Methods - CVSA classification Section) during the pre-cue resting period, i.e., from 1500 ms to 500 ms before the cue was displayed, for each trial. The IAP spectral distribution for each participant is provided in Fig. 13. Then, we computed the regression slope over all the trials to determine the evolution of this pre-cue *α*-power along the session. Finally, we performed 3 correlation analyses to assess the relationship between the obtained regression coefficients and the 3 metrics of CVSA performance and expertise described herein above.

## Data Availability

The datasets generated during and/or analysed during the current study are available from the corresponding author on request.
